# Protein identification from dried nipple aspirate fluid on Guthrie cards using mass spectrometry

**DOI:** 10.3892/mmr.2015.3432

**Published:** 2015-03-05

**Authors:** LUCAS DELMONICO, VIVIAN RABELLO AREIAS, RODRIGO CÉSAR PINTO, CINTIA DA SILVA MATOS, MARCO FELIPE FRANCO ROSA, CAROLINA MARIA DE AZEVEDO, GILDA ALVES

**Affiliations:** 1 Applied Genetics Laboratory, Haematology Service, National Cancer Institute, Rio de Janeiro, 20230-130, Brazil; 2 Medical Sciences Graduation Program, Medical Sciences Faculty, State University of Rio de Janeiro, Rio de Janeiro, 20550-170, Brazil; 3 Clinical Pathology Laboratory, National Cancer Institute, Rio de Janeiro, 20230-130, Brazil; 4 Radiology Service, Gaffrée e Guinle University Hospital, Rio de Janeiro, 20270-004, Brazil

**Keywords:** nipple aspirate fluid, breast neoplasms, proteome, mass spectrometry, Guthrie card

## Abstract

Nipple aspirate fluid (NAF) requires investigation as a potential source of biomarkers for early diagnosis or risk assessment in breast cancer and other breast disorders. The present study demonstrated that proteins were easily extracted from dried NAF spots on Guthrie cards and were suitable for mass spectrometry analysis. NAF was obtained from 80 women, collected on Guthrie cards, between 2007 and 2010. The NAF-proteins were extracted from the card by incubating the card in water. These proteins were then quantified and separated using one-dimensional, 12% SDS-PAGE, gel electrophoresis and on high-resolution gradient gels at different concentrations (4–12, 8–16 and 4–20%). The bands with the most abundant proteins were excised from the gradient gels and the proteins were identified by liquid chromatography quadrupole time of flight. Immunoglobulins, Zn-α2-glicoprotein, apoliprotein D and prolactin inducible protein were among those identified. The NAF-Guthrie card collection method has not been applied previously, however, NAF proteins have been identified using other collecting techniques, confirming the feasibility of the NAF Guthrie card collection method for analyzing the proteins within NAF. The NAF-Guthrie card collecting method has multiple advantages, including being inexpensive, non-invasive, reliable and painless, and the cards can be stored at room temperature. Examining NAF may assist in identifying individuals at a higher risk of breast cancer and in improving patient prognosis.

## Introduction

Breast cancer is the most commonly diagnosed type of cancer in female individuals, and 99% of the cases of breast cancer originate in the ductal and lobular epithelia (1,2). The breast cancer screening tools available at present, including mammography and careful palpation of the breasts do not detect up to 40% of early cases of breast cancer and are the least effective in detecting cancer in young females, whose tumors are often more aggressive (2). In addition, further invasive diagnostic methods, including needle aspiration or surgical biopsy, are required to determine whether the breast lesion is cancerous (3). Despite the use of mammography, breast cancer is often undetected at an early stage in Brazil (4). Thus, the development of a noninvasive and effective method to diagnose early-stage breast cancer is necessary to improve patient prognosis.

Nipple aspirate fluid (NAF) is continuously secreted and reabsorbed in non-pregnant and non-lactating women from the ductal and lobular system of the breast (5). NAF is a potential source of biomarkers for the early diagnosis or risk assessment of breast cancer, it is produced at all ages between puberty and the menopause, and can arise from malignant breast tumors and benign diseases, including inflammation, fibrocystic diseases and ductal ectasia (6).

As currently used techniques for the collection of NAF are either invasive or are pump-based, the application of Guthrie cards for collecting small quantities of NAF for subsequent protein extraction was investigated in the present study. Guthrie cards are routinely used for the collection of blood spots from heel prick assessements in newborns, to screen for metabolic disorders, including phenylketonuria. In addition, Guthrie cards are appropriate for protein conservation (7).

The present study aimed to investigate whether proteins can be extracted from dried NAF spots on Guthrie cards. In order to verify the feasibility of this method, a qualitative proteomics analysis was performed using liquid chromatography quadrupole time of flight (LC-Q-TOF) for certain proteins obtained by this method, which were separated in high-resolution gradient gels.

## Materials and methods

### Subjects

The Ethical Committees from the Gaffrée e Guinle University Hospital (Rio de Janeiro, Brazil) approved subject participation for the present study; written informed consent was obtained from all patients. Between May 2007 and December 2010, 88 eligible female individuals, ≥18 years old, were recruited with spontaneous nipple discharge at HUGG. Reasons for exclusion included pregnancy, lactation within the last 12 months, previous subareolar or other surgery, which may have disrupted the ductal systems and immunological deficiency by virus. Certain individuals had specimens analyzed from one breast only, whereas others had specimens analyzed from two breasts. In 50 females, NAF was secreted from one breast, whereas NAF was secreted from both breasts in 28 females. As for the origin of the discharge, certain females exhibited effusion from one orifice in the nipple, while others exhibited multiple orifices in the nipple, providing spontaneous NAF. Specimens were only collected when the NAF was easily obtainable. The NAFs were classified by their macroscopic characteristics, including whether they were watery, citrine, serous, bloody or mixed (seropurulent). All participants were subject to mammography and breast ultrasonography examinations and clinical evaluation.

### NAF collection

**Figure 1:**
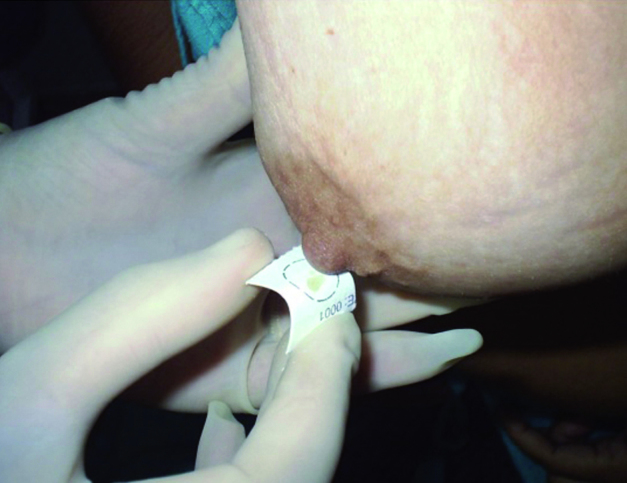
Collection of nipple aspirate fluid onto the Gurthrie card.

**Figure 2:**
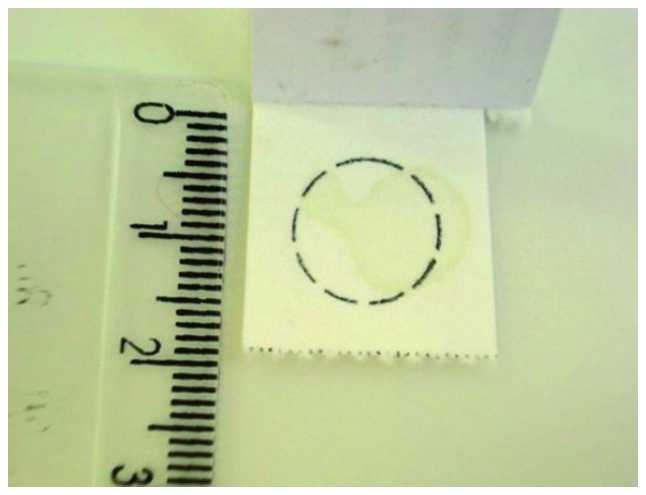
Nipple aspirate fluid spot absorbed onto the Guthrie card.

NAF was collected using a modification of a previously described technique (8,9). The participants had their breasts warmed for 10–30 min using bilateral hot compress pads wrapped in towels. The nipple was cleaned with alcohol and the breast was gently massaged from the chest wall toward the nipple for 1 min. Subsequently, the participant gently compressed the breast with two hands and the fluid, which appeared in the form of droplets, was collected onto Guthrie cards (GE Healthcare Bio-Sciences, Pittsburgh, PA, USA), as shown in Fig. 1 and were stored at room temperature (Fig. 2). Up to three attempts were made to obtain fluid on each breast. If no fluid appeared following the third attempt, the participant was considered a non-provider. The NAF-Guthrie cards used in the present study had been stored for a period of 2–4 years.

### Protein extraction and gels

The Guthrie spots were cut into sections of ~6 mm^2^ and each was incubated in 100 *μ*l double-distilled water for 30 min at 56°C. The soluble NAF proteins were mixed with 1 *μ*l phenylmethanesulfonyl fluoride (0.2 mg/ml; Sigma-Aldrich, St. Louis, MO, USA) and measured using the bicinchoninic acid or Smith reagent methods (Pierce BCA Protein Assay kit; Pierce Biotechnology, Inc., Rockford, IL, USA) (10).

The NAF proteins were separated by polyacrylamide gel electrophoresis using SDS-PAGE and β-mercaptoethanol (Sigma-Aldrich). The stacking gel was prepared at room temperature using 4% acrylamide (Sigma-Aldrich) Tris-HCl (0.5 M pH 6.8; Sigma-Aldrich), containing 0.4% SDS, and the separating gel was prepared at room temperature using 12% acrylamide Tris-HCl buffer (1.5 M, pH 8.8), containing 0.4% SDS (Sigma-Aldrich). The electrode buffer used was Tris-glycine (0.025 M Tris base and 0.192 M glycine; pH 8.3; All from Sigma-Aldrich), containing 0.1% SDS. Each sample (20 *μ*g of each) was mixed with the sample buffer to a final concentration of 0.06 M Tris-HCl pH 6.8, 2% SDS, 5% β-mercaptoethanol, 10% glycerol and 0.025% bromophenol blue. The samples were heated to 95°C for 3 min and loaded onto the gel in the mini-Protean II system (Bio-Rad Laboratories, Inc., Hercules, CA, USA), running at 39 mA/120V for 90 min at room temperature. The molecular weight standard was BenchMark Protein Ladder (Invitrogen Life Technologies, Carlsbad, CA, USA). Following electrophoresis, the gels were fixed for 1 h in a solution of 40% (v/v) aqueous ethanol (99.8%; Sigma-Aldrich) and 10% (v/v) acetic acid (Merck Millipore, Darmstadt, Germany) at room temperature. The gels were then washed for 30 min in fresh fixing solution and incubated with Coomassie Blue R-250 0.2% diluted in fixative solution for 2 h at room temperature (Sigma-Aldrich). The gels were destained using fixative solution for 2 h, followed by incubation in water at room temperature until complete destaining.

The NAF proteins were also separated using Amersham ECL high resolution gradient gels (GE Healthcare Life Sciences, Chalfont, UK), with concentrations of 4–12, 8–16 and 4–20%. Total protein (5 *μ*g) was added onto the gel with sample buffer 1:1 (50 mM Tris-HCl, pH 6.8, 2% SDS, 0.1% bromophenol blue and 10% glycerol). This system has a horizontal electrophoresis field and the gels comprise buffers, which improve the resolution of complex samples. The molecular weight standard used was Benchmark Protein Ladder (Invitrogen Life Technologies). Following electrophoresis, the gels were stained with Coomassie Blue R-250, according to the manufacturer’s instructions, and were analyzed for protein integrity and the to determine the profile of the revealed bands.

### Enzymatic digestion for nano(n)LC-Q-TOF

Selected bands, as described by Manello *et al* (11), were excised for destaining and were subjected to enzymatic digestion, according to Shevchenko *et al* (12) with modification of the destaining phase, where the bands were destained in a solution of 25 mM ammonium bicarbonate (pH 8.8/50%; Sigma-Aldrich) and acetonitrile (ACN) overnight in a shaker, at room temperature. All the samples were concentrated in a Speed-Vac Centrifuge (Thermo Fisher Scientific, Waltham, MA, USA) at 3,000 × g for 5 min to produce a 20 *μ*l final volume of digested ultrafiltrate sample (DIUs).

### Analysis of DIUs by nLC-Q-TOF

Prior to the nLC-Q-TOF analysis of the DIUs, they underwent manual desalination Zip Tip (Eppendorf, Hamburg, Germany). Each Zip Tip was activated with 10 *μ*l ACN (100%; Merck Millipore), was washed three times with 10 *μ*l ultrapure sterile water, and 10 *μ*l sample was loaded by pipetting up and down 10 times within the tube. Each Zip Tip was then washed three times using sterile ultrapure water, and ACN elution was performed. Subsequently, the samples were reduced to a final volume of 20 *μ*l and were stored at −20°C until analysis using mass spectrometry (Q-TOF Ultima Global; Waters, Manchester, UK).

The extracted peptides from the SDS-PAGE gel slice were loaded into an electrospray ionization quadrupole time-of-flight (ESI-Q-TOF) mass spectrometer (Waters Corporation, Wilmslow, UK). The DIU samples were loaded onto the Waters nanoACQUITY UPLC^®^ System (Waters Corporation, Milford, MA, USA), with a Waters Opti-Pak C18 trap column coupled to Q-Tof Ultima^®^ (Waters Corporation, Milford, MA, USA). Subsequently, 3.0 *μ*l sample was injected into a nanoEase C18 150 mM × 75 *μ*m column (Waters Corporation) at a flow rate of 0.6 *μ*l/min, and eluted with ACN containing 0.1% formic acid. The instrument control and data acquisition were performed using a MassLynx data system (Version 4.0, Waters Corporation). The experiments were performed by scanning from a mass-to-charge ratio (m/z) of between 200 and 2,000. The exact mass was automatically determined using the Q-Tof’s LockSpray™ (Waters Corporation, Milford, MA, USA).

### Database searching

The data were processed using ProteinLynx Global Server (version 2.0, Waters Corporation) for ESI-Q-TOF analysis. The proteins were identified by the correlation of tandem mass spectra to the NCBInr proteins and MSDB database, using MASCOT online software (www.matrixscience.com). The first analysis considered all taxonomies, while the second analysis was restricted to *Homo sapiens* to remove redundant protein identification.

### Breast imaging

Conventional mammography was performed in a Mediman HFG/B unit with Kodak min-R-2000 film and a Kodak RP-X-Omat processor (Kodak, Rochester, NY, USA). Ultrasonography was used as a complementary examination to the conventional mammography, and was performed using an Image Point Hx unit (HP Labs, Palo Alto, CA, USA), with two transducers (7.5 and 10 MHz) that measured the diameters of the breast ducts. The images were then classified using a breast imaging reporting and data system (BI-RADS).

## Results

**Figure 3:**
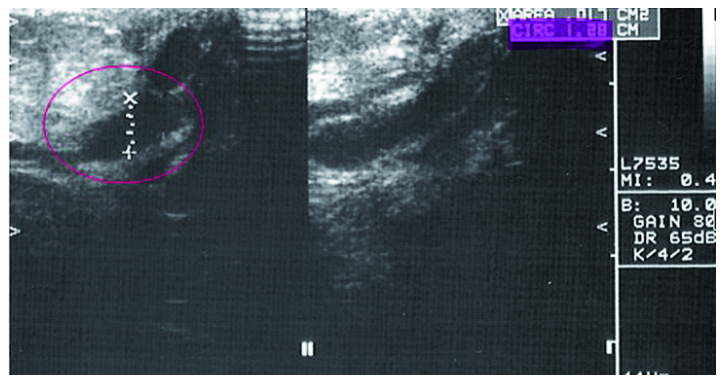
Ultrasonography revealing ductal ectasia on the left and right sides. The pink circle was added by the radiologist to emphasize the duct.

From the 88 female individuals enrolled in the present study, NAF was obtained from 80 (91%) on the first visit, which was collected and absorbed onto Guthrie cards, using the gentle massage and warming procedure. Of these 80 individuals, two were excluded due to subsequently identified immunological deficiency, and the remaining group was composed of 78 individuals, with a mean age of 50.24 years (range: 23–77 years) and menarche at a mean age of 13.24 years (range: 9–18 years). A total of 43 (55%) were postmenopausal. The mean age at menopause was 48.58 years (range: 36–54 years). A total of 64 became pregnant and of these, 11 did not breastfeed. Of the total group, 18 individuals (23%) had either an abortion (8; 44%) or a miscarriage (10; 66%). A total of 52 women (67%) reported a family history of cancer, however, only four reported a family history of breast cancer, of which there was only one confirmed case of hereditary breast cancer. The results of the mammography examinations assigned 73% of the individuals to the BI-RADS 0 category (inconclusive diagnosis), which required additional assessments, 46% of which had ultrasonography and 77% were identified as BI-RADS 3 (benign lesions). Ultrasound was performed, which revealed the predominance of ductal ectasias (Fig. 3). In addition, other injuries were observed, including the presence of nodules, axillary lymph nodes and microcalcification.

A total of 106 NAF spots were obtained on the Guthrie cards, which were characteristically classified into the following five types: Watery, citrine, serous, bloody and mixed (Table I). NAF was obtained from both breasts in 28 of the females, explaining why the total number of NAF spots was higher than the number of individuals enrolled. The NAF classification from both breasts were the same, with the exception of three cases.

**Figure 4:**
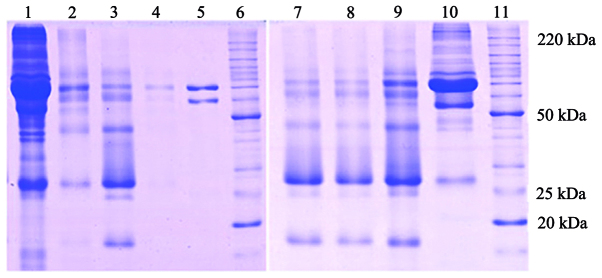
One-dimensional electrophoresis of the NAF proteins stained with Coomassie Brilliant Blue R-250. Lane 1, bloody NAF; lanes 2, 3 and 4 watery NAF; lane 5, bovine serum albumin; lanes 6 and 11 molecular weight; lanes 7, 8 and 9, mixed NAF; lane 10, citrine NAF. NAF, nipple aspirate fluid.

**Figure 5:**
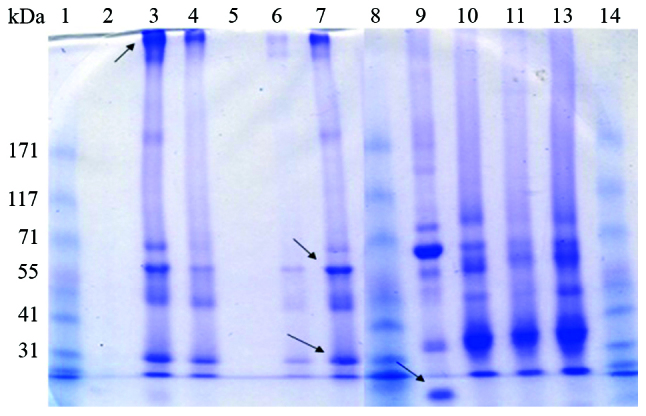
High resolution gradient gel 4-12% electrophoresis of the NAF proteins. Lanes 1, 8 and 13, molecular weight; lanes 2 and 5, blanks; lanes 3 and 7, bloody NAF; lanes 4 and 10, mixed NAF, lane 6, watery NAF; lane 9, citrine NAF; lanes 11 and 12, serous NAF. Arrows indicate the bands that were excised for subsequent proteomic analysis. NAF, nipple aspirate fluid.

The protein concentration of NAF ranged between 6.8 and 11.2 *μ*g/*μ*l, with a mean value of 9.2 *μ*g/*μ*l. Analysis of the NAF proteins was performed using one-dimensional SDS-PAGE 12% gel electrophoresis, which revealed five major bands, with each sample containing similar quantities of protein. Using the Guthrie card collection method, the proteins were found to have a similar band pattern as those described by Mannello *et al* and Varnum *et al* using an aspiration system (11,13). Differences from the default bands in the protein gel were classified according to band presence, absence and intensity variation (Fig. 4). No differences were observed in the bands in the watery and mixed NAF groups, compared to the higher molecular weight bands of the citrine group, between the gradient gels and the SDS-PAGE 12% gels. The 4–12% gel exhibited the highest resolution (Fig. 5) and was selected for use in the gradient gels. The greatest difference was confirmed in the bands <20 kDa. This difference requires further investigation, but were considered to be associated with cystic breast disease and benign breast lesions (13,14).

Of the bands excised, the spectra predominates were identified and the peptide score was calculated as −10Log(P), where P (0.05) is the probability that the observed match was a random event. Table II shows the major proteins that were identified with a score >50 in the nLC-Q-TOF. Immunoglobulins, Zn-α2-Glicoprotein, apoliprotein D and prolactin inducible protein were among the bands assessed. The NAF-Guthrie card collection has not been applied previously, however, NAF proteins have been identified using other collecting methods (11,15), confirming the feasibility of the NAF-Guthrie card collecting method.

## Discussion

Examining the breast epithelium directly using core needle biopsy or ductal lavage is uncomfortable and invasive. By contrast, the NAF-Guthrie card collecting method is inexpensive, non-invasive, reliable and painless. This method may broaden the applicability of NAF sample collection and may have an advantage over the method described by Sauter *et al* (16), which used an aspiration device. In addition, Guthrie cards occupy little space and can be stored at room temperature with dried NAF. These characteristics enable the cards to be sent to a laboratory for analysis.

Identifying associations in the results of imaging techniques is hindered by their own limitations in the public health system in Brazil (4), however, a significant association was observed between ductal ectasia and the secretion of NAF. In addition, the majority of the mammogram results were BI-RADS 0, which are flagged as abnormal due to the ability of non-palpable lesions to disturb results, leading to a false-negative diagnosis (17).

A low percentage of the female individuals selected for the present study were receiving hormone therapy or oral contraceptives (data not shown), which did not enable the investigation of associations between the secretion of NAF and these variables. The small 20 kDa protein, in particular the Gross cystic disease fluid protein, as described by Mannello *et al* is markedly associated with alterations in the breast (15,16). Human epidermal growth factor receptor-2 (HER2) is a breast cancer subtype biomarker, the amplification/overexpression of which is associated with aggressive disease and a poorer prognosis (18). In ductal carcinoma *in situ*, overexpression of HER has been observed by immunohistochemistry and is correlated with higher proliferative activity (19). HER2 has previously been detected in NAF, in addition to breast related hormones, metabolites and growth factors (18–24). Thus, it was suggested that breast cancer biomarkers can be detected using dried NAF spots in Guthrie cards, followed by mass spectrometric analysis. In the present study, gels were produced from the NAF proteins in order to characterize them; however, for clinical use, the shotgun mass spectrometry approach, with no in-between gel, is considered more appropriate.

In conclusion, the NAF-Guthrie card collecting method used in the present study, was confirmed as being suitable for modern mass spectrometric analysis. This method has potential application for early breast cancer screening and subtype classification.

## Figures and Tables

**Table I:** Classification of the breast fluid collected.

Type	Number	%
Watery	18	17
Citrin	5	5
Serous	35	33
Blood	12	11
Mixed (seropurulent)	36	34

**Table II:** List of the major proteins identified by liquid chromatography quadrupole time of flight.

Band position	Protein	Score (Ion score>50)	Mass (m/z)	Match (n)
15 (kDa)	Hemoglobin subunit β	1937	16102	59
	Hemoglobin subunit δ	760	16159	36
	**Prolactin-inducible protein**	523	16847	25
	**Apoliprotein D (fragment)**	219	15305	27
	Ig α-1 chain C region	214	38486	11
	Ig γ-1 chain C region	81	36596	3
	**Putative zinc-α-2-glycoprotein-like 1**	79	23080	4
	Immunoglobulin J chain (Fragment)	78	18509	2
20 (kDa)	Serum albumin	4942	71317	163
	**Apoliprotein D**	880	24541	40
	Clusterin (fragment)	147	33794	9
	**Prolactin-inducible protein**	132	16847	12
	α-1-Antitrypsin	54	46878	2
50 (kDa)	Ig α-1 chain C region	1483	38486	59
	Ig α-2 chain C region	1483	37301	62
	**Apoliprotein D**	777	24541	36
	Serum albumin	625	71317	23
	Ig heavy chain V-III region BRO	291	13332	9
	Ig heavy chain V-III region CAM	102	13773	6
	**Prolactin-inducible protein**	227	16847	15
	Complement C4 β chain	172	194351	3
	α-1-Antitrypsin	154	46878	9
	Ig γ-1 chain C region	140	36596	13
	Secretoglobin family 1D member 2	117	10260	6
	**Zinc-α-2-glycoprotein**	101	34465	7
	Polymeric immunoglobulin receptor	88	84429	7
	Vasorin	79	72751	2
	Ig κ chain C region	75	11773	6
	Ig γ-2 chain C region	68	36505	9
	Chromosome-associated kinesin KIF4A	54	141390	2
200 (kDa)	Serum albumin	1290	71317	60
	**Apoliprotein D (fragment)**	102	24541	8
	Cdc42 effector protein 4	53	30253	2

Bold font represents the most abundant proteins in the nipple aspirate fluid. Ion score indicates the reliability of the results. Ig, immunoglobulin.
